# Laparoscopic Enbloc Gastric Resection of A Giant Gastrointestinal Stromal Tumor (GIST)

**DOI:** 10.1002/ccr3.70242

**Published:** 2025-07-15

**Authors:** Sara Saeidi, Vincenzo Pappalardo, Andres Hanssen, Salomone Di Saverio

**Affiliations:** ^1^ Center for Obesity Research, Innovation and Education, Department of Surgery Hartford Hospital Hartford Connecticut USA; ^2^ Department of General and Endocrine Surgery University of Insubria Varese Italy; ^3^ Universidad Libre Seccional Barranquilla. Clínica Iberoamerica Barranquilla Colombia, Clínica Portoazul Barranquilla Colombia Barranquilla Colombia; ^4^ Department of General Surgery and Surgical Oncology Madonna del Soccorso Hospital San Benedetto del Tronto Italy; ^5^ Department of General and Transplant Surgery Sapienza University of Rome Rome Italy

**Keywords:** gastrectomy, gastric neoplasm, gastrointestinal stromal tumors, minimally invasive surgery

## Abstract

Minimally invasive laparoscopic resection can be a feasible and effective option for managing large, bleeding gastric GISTs, even in emergency settings with organ adherence. Careful surgical planning and follow‐up are essential to prevent recurrence, as GISTs may reoccur, highlighting the importance of ongoing monitoring.

Gastrointestinal stromal tumors (GISTs) are common mesenchymal tumors of the digestive tract [1]. R0 resection is the preferred treatment for gastric GISTs [2], but the feasibility of laparoscopic resection in emergency cases involving large bleeding tumors with adherence is unclear. This report describes the laparoscopic enbloc resection of a giant GIST in a 69‐year‐old man who presented with gastrointestinal bleeding (Video S1).

The tumor, originating from the posterior gastric wall and adhering to the pancreas, was removed with free margins. The patient had no recurrence after 6 months. This case supports the efficacy of minimally invasive surgery for large, bleeding GISTs in emergencies.

## Author Contributions

**Sara Saeidi:** conceptualization, data curation, formal analysis, investigation, methodology, writing – original draft, writing – review and editing. **Vincenzo Pappalardo:** conceptualization, data curation, formal analysis, investigation, writing – review and editing. **Andres Hanssen:** conceptualization, investigation, supervision, writing – original draft, writing – review and editing. **Salomone Di Saverio:** conceptualization, data curation, investigation, supervision, validation, writing – review and editing.

## Consent

Written informed consent was obtained from our patient who is included in the video vignette.

## Conflicts of Interest

The authors declare no conflicts of interest.

## Supporting information


**Video S1.** Laparoscopic enbloc resection of a giant bleeding gastrointestinal stromal tumor (GIST). The video demonstrates key steps of the minimally invasive procedure, including tumor dissection, resection from adjacent organs, and final specimen extraction. This technique highlights the feasibility of laparoscopic management for large bleeding GISTs in an emergency setting.

## Data Availability

The data generated or analyzed during this study are available from the corresponding author upon reasonable request. Due to patient confidentiality and privacy concerns, individual patient data cannot be made publicly available. However, aggregated data and relevant findings from the study will be shared to promote transparency and facilitate further research.
